# Associations of metal profiles in blood with thyroiditis: a cross-sectional study

**DOI:** 10.1007/s11356-022-23625-1

**Published:** 2022-10-20

**Authors:** Yaosheng Luo, Huixian Zeng, Yanshi Ye, Genfeng Yu, Cheng Song, Siyang Liu, Xingying Chen, Yuqi Jiang, Hualin Duan, Yue Li, Shengqing He, Zhi Chen, Lingling Liu, Yongqian Liang, Xu Lin, Heng Wan, Jie Shen

**Affiliations:** 1 Department of Endocrinology and Metabolism, Shunde Hospital of Southern Medical University (The First People’s Hospital of Shunde Foshan), Foshan, Guangdong China; 2 Medical Research Center, Shunde Hospital of Southern Medical University (The First People’s Hospital of Shunde Foshan), Foshan, Guangdong China; 3 Department of Endocrinology and Metabolism, The Third Affiliated Hospital of Southern Medical University, Guangzhou, Guangdong China

**Keywords:** Magnesium, Iron, Autoimmune thyroiditis, TPOAb, TgAb

## Abstract

Autoimmune thyroiditis (AIT) is increasingly common, and serological markers include thyroid peroxidase antibody (TPOAb) and thyroglobulin antibody (TgAb). To determine if selected metals influence thyroiditis antibody positivity, this cross-sectional study investigated associations between metals and thyroiditis antibody status. Healthy individuals (*n* = 1104) completed a questionnaire and underwent checkups of anthropometric parameters, thyroid function status, and levels of seven metals in blood (magnesium, iron, calcium, copper, zinc, manganese, and lead). Associated profiles of glyco- and lipid metabolism were also established. Logistic regression and restricted cubic spline (RCS) regression analysis were applied to adjudge associations between metals and TPOAb and TgAb status. It was found that, after adjusting for likely cofounding factors, participants with antibody positivity had significantly lower serum concentrations of magnesium and iron. When serum magnesium levels were analyzed in quartiles, the odds ratios of quartile 4 were 0.329–fold (95% confidence interval (CI): 0.167–0647) and 0.259-fold (95% CI 0.177–0.574) that of quartile 1 regarding TPOAb and TgAb positivity (*P* = 0.004, 0.003). After adjustment, the RCS analysis detected nonlinear associations between iron and TPOAb and TgAb positivity (*P* < 0.01, both). In stratified analyses, these associations regarding magnesium and iron remained for women of reproductive age, but not for postmenopausal women and men. We conclude that lower serum levels of magnesium and iron are associated with incremental positivity of thyroiditis antibodies and may be among the most important metals contributing to AIT in women of reproductive age.

**Supplementary Information:**

The online version contains supplementary material available at 10.1007/s11356-022-23625-1.

## Introduction

Autoimmune thyroiditis (AIT) is a common autoimmune thyroid disease, and the main cause of hypothyroidism. It is considered a risk factor of miscarriage, prematurity (Korevaar et al. 2013), coronary heart disease (Chen et al. 2015), and thyroid cancer (Chen et al. 2013). AIT is characterized by the destruction of thyroid follicles and infiltration of lymphocytes. The serological markers of AIT are thyroid peroxidase antibody (TPOAb) and thyroglobulin antibody (TgAb) (Ralli et al. 2020). Thyroiditis antibody positivity is a common diagnostic marker of AIT.

The global prevalence of AIT has been increasing for decades. In China, 14.2% of the population are positive for thyroid antibodies, according to recent national research (Li et al. 2020). Rather than genetics, this high rate is due more to environmental factors, including chemical elements (Ott et al. 2011).

Trace elements are necessary for thyroid hormone synthesis, and nonmetallic elements such as iodine and selenium are pivotal to thyroid autoimmunity (Kohrle et al. 2005; Teng et al. 2006). However, little is known of the functions of metal elements. Magnesium (Mg) and calcium (Ca) significantly effect regulation of the immune system by forming ion channels (Feske et al. 2015). Erdal et al. (Erdal et al. 2008) suggested that patients with AIT had higher serum Mg levels and lower serum iron (Fe), compared with healthy controls. Stojsavljević and colleagues (Stojsavljevic et al. 2020) reported inverse correlations between AIT risk and serum zinc (Zn) and copper (Cu), respectively, but a positive correlation with serum manganese (Mn). Nevertheless, another study indicated no significant differences between AIT and healthy people regarding metals (Przybylik-Mazurek et al. 2011).

A consideration is that exposure to heavy metals, such as lead (Pb), tends to trigger diseases even at low levels (Sobhanardakani et al. 2018). The abnormal accumulation of Pb in the body contributes to the death of thyroid follicular cells and excessive oxidative stress, which is an etiological factor of AIT (Nie et al. 2017). Furthermore, a competitive association between heavy metals and trace elements easily leads to thyroid dysfunction.

We suspect that inadequate intake of metals increases the risk of AIT, and the present study helped to inform this hypothesis. Studies that have focused on associations between metals and thyroid autoimmunity are rare, and data is limited by insufficient sample sizes or a single element. To help determine if selected metals influence thyroiditis antibody positivity, the current cross-sectional study investigated respective associations between levels of Mg, Fe, Ca, Cu, Zn, Mn, and Pb in blood with thyroiditis antibody status in healthy subjects, located in Foshan, Guangdong, China.

## Methods

The Ethics Committee of Shunde Hospital of Southern Medical University approved the study protocol (20211103), which conformed to the ethical guidelines of the 1975 Declaration of Helsinki. All participants provided informed consent prior to enrollment.

### Study design and volunteer enrollment

This study was designed as a cross-sectional investigation and is registered at www.chictr.org.cn (ChiCTR2100054130). Volunteers were enrolled in 2021 through stratified random sampling from a healthcare center in Lecong, Shunde District, Foshan, China. For inclusion, participants were aged 18 to 80 years, a resident in Shunde for more than half a year, and not pregnant. From 1111 potential participants, 7 individuals were excluded due to an unsatisfactory blood sample that failed to reach the loading volume for the test. Finally, the study comprised 1104 subjects, with 468 and 636 men and women, respectively.

For analysis and comparisons, participants were separated into 4 groups based on serological antibody status: TPOAb^–^, TPOAb^+^, TgAb^–^, and TgAb^+^.

### Measurements

Trained study personnel administered a standard questionnaire to collect information on lifestyle characteristics and medications. Anthropometric parameters included body weight and height and were measured in accordance with a standard protocol. Body mass index (BMI) was calculated as weight in kilograms divided by height in meters squared (kg/m^2^). To avoid interference with circadian rhythm, participants were asked to fast, and blood samples were taken between 8:00 AM and 10:00 AM. All samples were shipped under cold chain management to a central laboratory (certified by the College of American Pathologists) and centrifuged within 4 h.

Levels of metals in blood were measured by inductively coupled plasma mass spectrometer (ICAP-RQ, Thermo Fisher Scientific, Waltham, USA). Thyroid function was tested by chemiluminescence immunoassay (Centaur XPT, Siemens, Erlangen, Germany). TPOAb was assessed using a UniCel Dxl800 (Beckman, Brea, USA), and TgAb with a Biolumi 8000 (SNIBE, Shenzhen, China). Blood lipid profiles were conducted by BS800 (Mindray, Shenzhen, China) and included the following: total cholesterol (TC), triglyceride (TG), high density lipoprotein (HDL), and low-density lipoprotein (LDL). Glycated hemoglobin (HbA1c) was measured with an HLC-723G8 (TOSOH, Tokyo, Japan).

### Outcome definitions

Current smoking was defined as smoking at least 100 cigarettes over a lifetime and currently smoking. Excessive alcohol consumption was considered alcohol intake ≥ 210 g (≥ 140 g) per week by men (women) as previous mentioned (Wan et al. 2022). Iodized salt intake was defined as using iodized salt consistently for more than a year. The normal reference ranges for thyroid function were as follows: free triiodothyronine (FT3) 3.28 to 6.47 pmol/L; free thyroxine (FT4) 7.64 to 16.0 pmol/L; and thyrotropin (TSH) 0.490 to 4.91 mIU/L.

TPOAb^+^ was considered TPOAb > 30.0 IU/mL, and TgAb^+^ as > 4.00 IU/mL. Normal reference ranges of the selected metals in blood were set as previously reported (Zeng et al. 2022): Ca, 56.8 to 76.0 mg/L; Cu, 749 to 1395 μg/L; Fe, 421 to 660 μg/L; Mg, 26.9 to 49.4 mg/L; Mn, 6.60 to 21.6 μg/L; Pb < 100 μg/L; and Zn, 5.00 to 7.50 mg/L.

### Statistical analysis

The data regarding features of the study participants were summarized as mean ± standard deviation or median (interquartile range [IQR]) for continuous variables and frequencies for categorical variables. Student’s *t* test, the Mann–Whitney U test, and the chi-squared test were used to calculate the differences between groups. Univariate and multiple logistic regression analyses were conducted to identify potential risk factors, which considered continuous data for age, BMI, TG, and TC and binary data for gender (male and female), alcohol consumption (abused and non-abused), smoking (yes and never), and iodized salt (yes and no). Odds ratios (ORs) and 95% confidence intervals (95% CIs) were calculated via the logistic regression model. A two-sided *P* value < 0.05 was considered statistically significant. All data were analyzed using SPSS Statistics (V21.0, IBM corporation, USA). Restricted cubic spline regression (RCS) analysis was performed with R software (V4.1.2).

## Results

### General characteristics of volunteers with or without thyroid antibody positivity

The 1104 subjects were apportioned to 4 groups based on thyroid antibody status, with populations of 919, 185, 953, and 151 individuals, respectively, in the TPOAb^–^, TPOAb^+^, TgAb^–^, and TgAb^+^ categories (Table 1). The mean age of the overall study population was 49.8 years, and the mean BMI was 24.0 kg/m^2^. Compared with the antibody-negative subgroup, the antibody-positive subgroup had a higher percentage of women and higher serum TSH level. The higher percentage of women, and higher TSH level, were more pronounced in the TPOAb^+^ groups compared with the TgAb^+^. In addition, the FT4 level was significantly lower in the TPOAb^+^ compared with the TPOAb^–^, but not in the TgAb^+^ relative to the TgAb^–^. For each of the metabolic profiles (TG, TC, HDL, LDL, and HbA1c%), there were no statistically significant differences among the antibody-positive and antibody-negative subgroups.

**Table 1:** General characteristics of the participants in the study

Features	General	TPOAb^−^	TPOAb^+^	*P*	TgAb^−^	TgAb^+^	*P*
N	1104	919	185	-	953	151	-
Age, year	49.8 ± 14.4	50.1 ± 14.5	48.5 ± 13.8	0.181	49.5 ± 14.5	51.5 ± 13.9	0.125
Gender (male, %)	468 (42.4)	414 (45.0)	54 (29.2)	< 0.001	416 (43.7)	52 (34.4)	0.033
BMI, kg/m^2^	24.0 ± 3.59	24.1 ± 3.63	23.5 ± 3.30	0.058	24.0 ± 3.61	24.0 ± 3.44	0.929
HbA1c, %	5.85 ± 0.913	5.86 ± 0.909	5.81 ± 0.936	0.480	5.84 ± 0.879	5.91 ± 1.10	0.405
TG, mmol/L	1.23 (0.880, 1.83)	1.24 (0.880, 1.88)	1.15 (0.860, 1.51)	0.137	1.24 (0.880, 1.88)	1.15 (0.860, 1.51)	0.426
TC, mmol/L	5.45 ± 1.15	5.48 ± 1.14	5.30 ± 1.16	0.061	5.45 ± 1.15	5.42 ± 1.11	0.714
HDL, mmol/L	1.41 ± 0.321	1.42 ± 0.316	1.41 ± 0.346	0.937	1.41 ± 0.319	1.41 ± 0.337	0.703
LDL, mmol/L	3.25 ± 0.941	3.26 ± 0.936	3.14 ± 0.941	0.110	3.23 ± 0.936	3.25 ± 0.952	0.790
Smoking, %	20.4	21.6	15.0	0.052	20.7	18.9	0.648
Alcohol, %	7.17	7.26	6.71	0.803	7.67	3.91	0.124
TSH, uIU/mL	1.59 (1.08, 2.40)	1.54 (1.06, 2.31)	1.80 (1.26, 2.81)	0.040	1.58 (1.08, 2.37)	1.69 (1.08, 2.49)	0.135
FT4, pmol/L	11.3 (10.2, 12.3)	11.4 (10.3, 12.3)	10.8 (10.0, 12.2)	0.018	11.35 (10.3, 12.3)	10.85 (9.91, 12.1)	0.020
FT3, pmol/L	5.46 (5.01, 5.95)	5.49 (5.03, 5.98)	5.42 (4.92, 5.86)	0.370	5.47 (5.02, 5.95)	5.42 (4.93, 5.96)	0.734
TPOAb positivity, %	16.8	0.000	100	< 0.001	8.80	66.9	< 0.001
TgAb positivity, %	13.7	5.40	54.6	< 0.001	0.000	100	< 0.001
Iodized salt, %	89.1	88.9	77.1	0.673	88.8	91.2	0.485
Ca, mg/L	63.0 ± 5.83	62.9 ± 5.85	63.6 ± 5.75	0.129	63.0 ± 5.69	63.4 ± 6.67	0.400
Mg, mg/L	41.7 ± 4.46	42.0 ± 4.35	40.5 ± 4.78	< 0.001	42.0 ± 4.36	40.4 ± 4.83	< 0.001
Mn, μg/L	13.1 ± 4.04	13.0 ± 3.86	13.6 ± 4.82	0.158	13.1 ± 3.88	13.4 ± 4.94	0.370
Fe, mg/L	505 ± 57.3	510 ± 54.0	481 ± 66.4	< 0.001	508 ± 53.8	487 ± 73.2	< 0.001
Cu, μg/L	886 ± 125	885 ± 123	893 ± 135	0.435	885 ± 124	890 ± 129	0.675
Zn, mg/L	6.32 ± 0.932	6.36 ± 0.915	6.09 ± 0.985	< 0.001	6.35 ± 0.924	6.11 ± 0.961	0.004
Pb, μg/L	20.0 (15.0, 27.0)	20.0 (16.0, 27.0)	19.0 (14.0, 25.0)	0.090	20.0 (15.0, 27.0)	20.0 (15.0, 26.0)	0.520

Continuous variables with normal distribution were presented as mean ± SD, and with skewed distribution presented as median (interquartile ranges), respectively. Categorical variables were summarized as a numerical proportion. *P* values were calculated by Student’s t test, Mann–Whitney U test, χ^2^ test. *P* < 0.05 was considered as significant different

BMI, body mass index; TPOAb, thyroid peroxidase antibodies; TgAb, thyroglobulin antibodies; TG, triglyceride; TC, total cholesterol; HDL, high-density lipoprotein; LDL, low-density lipoprotein; TSH, thyrotropin; FT3, free triiodothyronine; FT4, free thyroxine

Subjects who were antibody-negative showed significantly higher serum levels of Mg, Fe, and Zn compared with the antibody-positive groups (all, *P* < 0.001). Specifically, serum Mg levels were 42.0 ± 4.35 mg/L (40.5 ± 4.78 mg/L) in the TPOAb^–^ (TPOAb^+^) (*P* < 0.001) and 42.0 ± 4.36 mg/L (40.4 ± 4.83 mg/L) in the TgAb^–^ (TgAb^+^). The serum Fe levels showed similar results, with 510 ± 54.0 mg/L (481 ± 66.4 mg/L) in the TPOAb^–^ (TPOAb^+^), and 508 ± 53.8 mg/L (487 ± 73.2 mg/L) in the TgAb^–^ (TgAb^+^).

### Association of metals in blood and thyroid antibodies

To investigate the association between the metal concentrations in blood and thyroid antibodies, a logistic regression analysis was conducted of each element, based on model 1 without adjustment and model 2 with adjustments for covariates (Table 2). The analysis of possible relatedness between serum Mg, Fe, and Zn and TPOAb positivity showed ORs of 0.926, 0.992, and 0.725, respectively (95% CIs: 0.894–0.960, 0.989–0.994, 0.625–0.914; all *P* < 0.001). Similar results were observed for TgAb positivity.

**Table 2:** Association between metal levels and TPOAb and TgAb

TPOAb	Model 1		Model 2	
	OR (95%CI)	*P*	OR (95%CI)	*P*
Ca	1.02 (0.994, 1.05)	0.129	0.988 (0.948, 1.03)	0.566
Mg	0.926 (0.894, 0.960)	< 0.001	0.911 (0.864, 0.961)	0.001
Mn	1.03 (0.994, 1.07)	0.102	0.980 (0.928, 1.034)	0.461
Fe	0.992 (0.989, 0.994)	< 0.001	0.990 (0.986, 0.995)	< 0.001
Cu	1.00 (0.999, 1.00)	0.435	1.00 (0.999, 1.00)	0.675
Zn	0.725 (0.625, 0.914)	< 0.001	0.845 (0.667, 1.07)	0.162
Pb	0.976 (0.958, 0.995)	0.015	0.977 (0.947, 1.01)	0.138
TgAb	Model 1		Model 2	
	OR (95%CI)	*P*	OR (95%CI)	*P*
Ca	1.01 (0.985, 1.04)	0.344	0.985 (0.940, 1.03)	0.537
Mg	0.923 (0.888, 0.960)	< 0.001	0.879 (0.827, 0.934)	< 0.001
Mn	1.02 (0.981, 1.07)	0.287	0.988 (0.930, 1.05)	0.705
Fe	0.994 (0.991, 0.997)	< 0.001	0.993 (0.989, 0.998)	0.001
Cu	1.00 (0.999, 1.00)	0.675	1.00 (0.998, 1.00)	0.636
Zn	0.756 (0.625, 0.914)	0.004	0.956 (0.736, 1.24)	0.735
Pb	0.994 (0.976, 1.01)	0.503	1.01 (0.973, 1.04)	0.776

Model 1, unadjusted; Model 2, adjusted for age, gender, BMI, HbA1c, TG, TC, alcohol consumption, current smoking, and iodized salt intake

OR, odd ratio; CI, confidence interval. BMI, body mass index; TPOAb, thyroid peroxidase antibodies; TgAb, thyroglobulin antibodies; TG, triglyceride; TC, total cholesterol; HDL, high-density lipoprotein; LDL, low-density lipoprotein; TSH, thyrotropin; FT3, free triiodothyronine; FT4, free thyroxine

In model 2 the covariates for adjustments were age, gender, BMI, HbA1c, TG, TC, alcohol consumption, current smoking status, and iodized salt intake (Table 2, right). After adjustments, the associations between Mg and Fe and TPOAb positivity were indicated by ORs of 0.911 and 0.990, respectively (95% CIs: 0.864–0.961, and 0.986–0.995; both *P* < 0.001). In addition, when TgAb positivity was considered as a dependent variate, Mg and Fe showed ORs of 0.879 and 0.993 (95% CI: 0.827–0.934 and 0.989–0.998; *P* < 0.001, 0.01). However, Zn was found with no statistically difference after adjustment.

Interestingly, in the stratified analysis by gender, associations between Fe and Mg and antibody positivity were significant in women of reproductive age, but not in subgroups of postmenopausal women or men after adjustment. Subjects with low levels of Mg had higher rates of TPOAb positivity (OR 0.827 [95% CI 0.746–0.917]) and higher rates of TgAb positivity (OR 0.791 [95% CI 0.696–0.899]). The results for Fe were similar, with ORs of 0.984 (95% CI 0.977–0.992) for TPOAb and 0.982 (95% CI 0.973–0.991) for TgAb, all *P* < 0.001 (Supplemental Table 1).

### Association between Mg and thyroid immunity

**Fig. 1:**
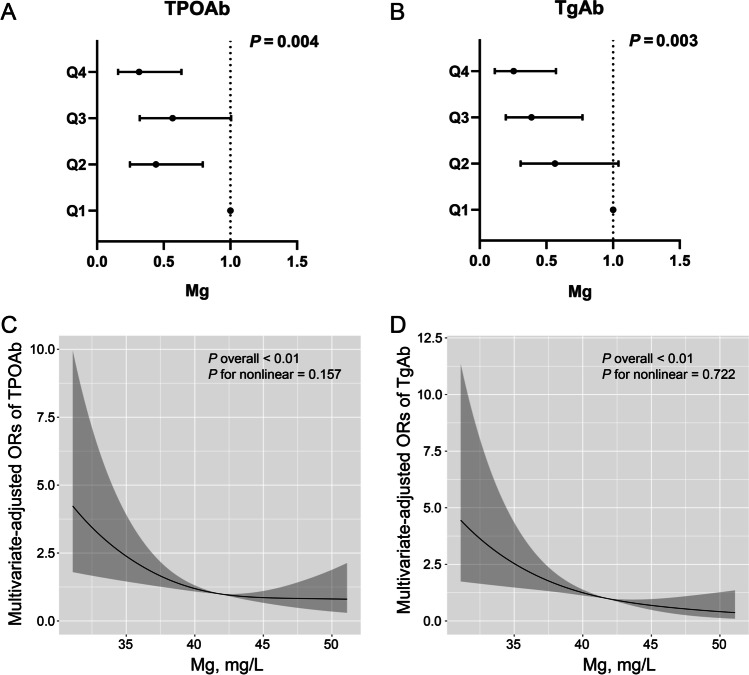
Association between magnesium and thyroid antibodies. **A** Mg quartiles and TPOAb by logistic regression analysis; **B** Mg and TgAb by logistic regression analysis; **C** Mg and TPOAb by RCS; **D** Mg and TgAb by RCS. All analyses were adjusted for age, gender, BMI, smoking, drinking, HbA1c, iodized salt intake, TG, TC. BMI, body mass index; TPOAb, thyroid peroxidase antibodies; TgAb, thyroglobulin antibodies; TG, triglyceride; TC, total cholesterol; HbA1c, glycated hemoglobin; RCS, restricted cubic spline regression analysis

Mg levels were separated into quartiles (Supplemental Table 2). The following were significantly higher in quartile 4, compared with quartile 1: percentage of men, BMI, TG, TC, LDL, percentage of smokers, percentage of alcohol drinkers, FT3; but lower in TPOAb positivity and TgAb positivity. After adjusting for age, gender, BMI, HbA1c, drinking, smoking, and iodized salt intake, the TPOAb positivity of quartile 4 was 32.9% of quartile 1 (OR 0.329; 95% CI 0.167–0.647; *P* = 0.001; for TgAb positivity, quartile 4 showed an OR 0.259 (95% CI 0.117–0.574; Fig. 1). A linear association was found among the 4 quartiles by a gradual reduction of the ORs, from quartile 1 to quartile 4, which was supported by the RCS analysis, with *P* values for nonlinearity of 0.157 and 0.722 for TPOAb and TgAb, respectively (Fig. 1, C&D). Associations were also adjusted for the covariates mentioned above.

### Association between Fe and thyroid immunity

**Fig. 2:**
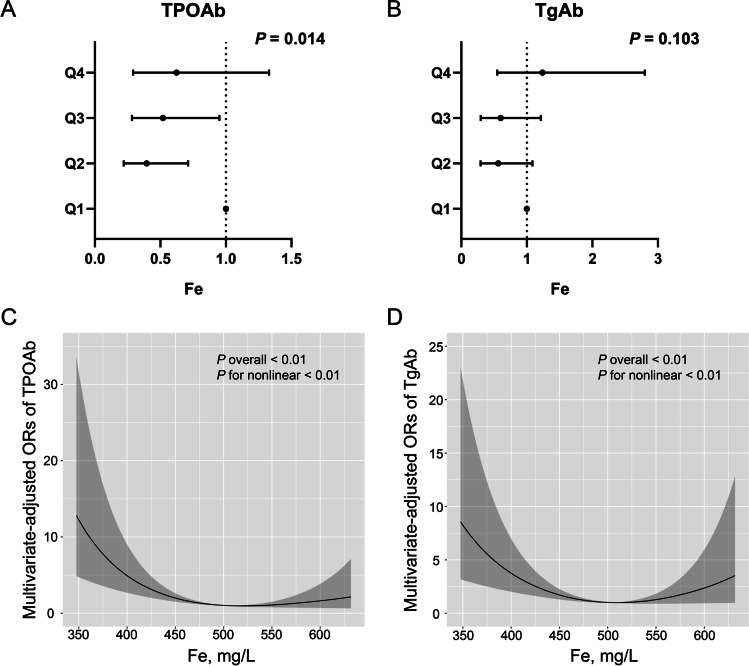
Association between iron and thyroid antibodies. **A** Fe quartiles and TPOAb by logistic regression analysis; **B** Fe and TgAb by logistic regression analysis; **C** Fe and TPOAb by RCS; **D** Fe and TgAb by RCS. All analyses were adjusted for age, gender, BMI, smoking, drinking, HbA1c, iodized salt intake, TG, TC. BMI, body mass index; TPOAb, thyroid peroxidase antibodies; TgAb, thyroglobulin antibodies; TG, triglyceride; TC, total cholesterol; HbA1c, glycated hemoglobin; RCS, restricted cubic spline regression analysis

Fe was also divided into quartiles (Supplemental Table 3). The following were higher in quartile 4, compared with quartile 1: percentage of men, BMI, TG, TC, LDL, percentage of smokers, percentage of alcohol drinkers and FT3. However, significant reductions were shown for age, HDL, TSH level and TPOAb positivity. For TPOAb, the logistic analysis showed that, for Fe, the ORs of quartiles 2 and 3 were lowered, respectively, to 0.407 (95% CI 0.227–0.729) and 0.528 (95% CI 0.294–0.952), but quartile 4 was not statistically different than quartile 1 (Fig. 2). For TgAb, there were no significant differences among the Fe quartiles. The RCS analysis after adjustment indicated nonlinear associations between Fe and TPOAb and TgAb positivity (*P* for nonlinearity < 0.01, both).

## Discussion

This study investigated associations between thyroid autoimmunity and blood metal element profiles (Mg, Fe, Ca, Cu, Zn, Mn, and Pb) in a community-based population. It was found that Mg and Fe were, respectively, and significantly inversely correlated with serum TPOAb and TgAb levels, especially in women of reproductive age, after corrections for age, gender, BMI, smoking status, alcohol intake, iodized salt intake, HbA1c, TG, and TC. In addition, logistic regression and RCS analyses showed that serum Fe correlated with TPOAb/TgAb positivity in a nonlinear manner, while Mg linearly correlated with these thyroiditis-associated antibodies. These unexpected results indicate that monitoring serum metal levels and adequate supplementation of Mg and Fe may be beneficial for prevention of AIT, especially for women of reproductive age.

The diagnostic sensitivities of TPOAb and TgAb for AIT are about 85% to 90%. Both of them have high specificity in humans and high affinity to their antigen epitopes (Beever et al. 1989), albeit low titer can be found in other autoimmune diseases (Barker et al. 2005). Patients with AIT tend to be positive for both TPOAb and TgAb, which is consistent with the data of the present cohort. Furthermore, upon stimulation, these two antibodies fluctuate in a parallel manner. The prevalence of positive TPOAb and TgAb in the present study was 16.8% and 13.7%, respectively, which is comparable to previous data in China (Li et al. 2020).

Compared to TgAb, TPOAb is considered the better marker of AIT. The titer of TPOAb closely correlates to lymphocyte infiltration in the thyroid gland and abnormal thyroid function (Ralli et al. 2020). Thyroid follicular cells are damaged by TPOAb via antibody-dependent cell-mediated and complement-dependent cytotoxicity and the complement system (McLachlan and Rapoport 2000). Unlike TPOAb, TgAb is often associated with fibrosis of the thyroid. Elevated TgAb tends to be more readily observed in mouse models of AIT. To some extent, TgAb correlates with thyroid cancer (Kim et al. 2010, Spencer and Fatemi 2013).

Serum Mg is a common measure of Mg status in humans (Costello et al. 2016), although it accounts for less than 1% of the Mg in the body as the majority is stored intracellularly (Grober et al. 2015). Deficiency is more common than overload, the latter often results in renal failure. Previous studies reported that serum Mg level correlates with the rate of hypothyroidism (Al-Hakeim 2009; Wang et al. 2018a). The production of thyroid hormones depends on the sodium-iodine symporter via active transport, a process that relies on oxidative phosphorylation and ATP synthesis (Tyler et al. 1968). Mg, a key enzyme cofactor in mitochondrial oxidative phosphorylation, importantly affects energy supply (Grober et al. 2015). In rat experiments, radioactive iodine uptake by thyroid follicular epithelial cells was significantly higher in rats given Mg supplementation (Humphray and Heaton 1972).

However, the association between Mg and AIT is still poorly understood. The present data indicates that serum Mg was significantly lower in people who were TPOAb^+^ or TgAb^+^, relative to those with TPOAb^–^ or TgAb^–^, although still within the normal reference range. After adjustments, Mg remained inversely correlated with thyroid antibody positivity. Similar results were found in another Chinese study, where severely low levels of Mg were associated with clinical TgAb positivity (Wang et al. 2018a). In addition, Klatka et al. (Klatka et al. 2013) found that serum Mg negatively correlated with activation of lymphocytes in Graves’ disease, indicating that thyroid-associated immune tolerance was impaired at low Mg levels.

Mg may act as an important regulator of immunity. Hypomagnesaemia appears to be associated with increased pro-inflammatory cytokine secretion. Mutations of MAGT1 (magnesium transporter 1), a Mg-specific transporter, contributes to dysfunction of T cells and NK cell activation (Chaigne-Delalande et al. 2013). In addition, Mg promotes LFA-1 (lymphocyte-function-associated-antigen-1), a co-stimulatory molecule on the membranes of CD8^+^ T cells which has antitumor properties (Lotscher et al. 2022). More studies are needed of the underlying mechanism between Mg and autoimmunity.

The present study also found that Fe could act as an independent protective factor, as serum Fe concentration was lower in antibody-positive patients, compared to antibody-negative patients. Fe homeostasis is important for thyroid function as well. Insufficient Fe leads to impaired thyroid hormone synthesis and interferes with the conversion of T4 thyroid hormone to T3 (Fayadat et al. 1999). Furthermore, Fe deficiency was a risk factor of AIT, especially those with thyroid dysfunction (Khatiwada et al. 2016). Erdal et al. (Erdal et al. 2008) found that low serum Fe concentrations in AIT improved after levothyroxine supplementation. A meta-analysis suggested that Fe deficiency can increase the risk of TPOAb positivity, especially in women of reproductive age (Luo et al. 2021). This is consistent with the present result.

The present study showed that serum Fe level affected TPOAb positivity more so than TgAb. A probable explanation is that TPO acts as a hemoglobin-containing enzyme that has Fe in its active center (Fayadat et al. 1999). Previous investigations revealed that AIT often occurs concurrently with other autoimmune diseases, including celiac disease and autoimmune gastritis (Pinto-Sanchez et al. 2015), and Fe deficiency is frequent. Fe is also important for the activation of immune cells, such as macrophages (Soares and Hamza 2016). In the present study, logistic regression and RCS analyses indicated that among all the metals analyzed, only adequate Fe level may be preventive.

Yet, an overload of Fe can lead to nervous system injury. Ferrous iron ions may generate toxic reactive oxygen species, leading to harmful oxidation of lipids and DNA, cell damage, and inflammation (Kell 2009). Iron deposition can also stimulate the development of autoimmune diseases (Wang et al. 2018b). Wang and colleagues (Wang et al. 2018b) reported that iron accumulation promoted pro-inflammatory cytokine production via RNA-binding protein sensing. Therefore, we recommend that iron supplementation, after fulfilling iron storage, should be cautiously applied.

The gender-stratified analysis indicated that the associations mentioned above were also true for women of reproductive age. The subgroup analysis showed no statistical differences between men and postmenopausal women, but compared with these groups, women of reproductive age have a higher prevalence of autoimmune diseases. A possible explanation may be an association between menopausal cycle and element homoeostasis, as Fe is lost through menstrual blood (Yokoi 2014). Other research has also supported that Fe level is associated with the cycle of reproductive hormones (Badenhorst et al. 2021). These data suggest that Fe deficiency occurs commonly in women of reproductive age and contributes to triggering AIT.

In this study, the serum levels of other metals (Zn, Ca, Mn, Cu) and blood Pb were also assessed and found to support thyroid homeostasis. Zn in the thyroid gland is important for thyroid function and the immune system (Stojsavljević et al. 2020). However, in the present study, after adjusting for confounding factors, there was no difference among the antibody groups, which was particularly noteworthy for BMI. Cayir et al. (Cayir et al. 2014) reported that the serum Zn levels of children with exogenous obesity were significantly lower than that of a healthy control group, suggesting that alterations in zinc intake due to a diet leading to obesity may cause detrimental changes in serum thyroid hormones. Stojsavljević et al. (Stojsavljevic et al. 2020) found that heavy metal elements were elevated in the thyroid glands of patients with AIT. The SPECT-China study also reported that Pb negatively influenced AIT in women (Nie et al. 2017). However, our data suggested no significant difference in Pb, although this may be due to sample size and ethnic bias.

There are some limitations in this study. Firstly, as a cross-sectional study, causality between Mg or Fe with thyroid antibodies could not be confirmed. Future studies may focus on longitudinal observation and mechanism interpretation. Secondly, the major participants live in a coastal area of China characterized by adequate iodine-intake, which affects basic thyroid function, and so the results may be biased due to ethnicity and location. Thirdly, interactions among the elements may have confounded the multivariate approach.

## Conclusion

This study determined that, among healthy subjects, those with low serum Mg and Fe levels were more likely to have thyroiditis-associated antibodies. After adjustments, Mg showed an inverse linear association, while that of Fe was nonlinear. Monitoring metal levels in blood and adequate supplementation of Mg and Fe may be beneficial for thyroid homeostasis, especially for women of reproductive age.

## Supplementary Information

Below is the link to the electronic supplementary material.

Supplementary file1 (DOCX 27 KB)

## Data Availability

The data supporting the findings of the study are available from the corresponding authors upon reasonable request.

## Abbreviations

AIT: Autoimmune thyroiditis
BMI: Body mass index
CI: Confidence intervals
FT3: Free triiodothyronine
FT4: Free thyroxine
HbA1c: Glycated hemoglobin
HDL: High-density lipoprotein
LDL: Low-density lipoprotein
QR: Interquartile range
OR: Odds ratio
RCS: Restricted cubic spline regression analysis
SD: Standard deviation
TC: Total cholesterol
TEC: Thyroid follicular cells
TG: Triglyceride
TgAb: Thyroglobulin antibodies
TPOAb: Thyroid peroxidase antibodies
TSH: Thyrotropin
