# Supplementary material for: Associations of metal profiles in blood with thyroiditis: a cross-sectional study
Source: Environ Sci Pollut Res Int. 2022 Oct 20;30(8):21072–80. doi: 10.1007/s11356-022-23625-1 (PMC9938027; doi:10.1007/s11356-022-23625-1)
Supplement: Supplementary file 1 — Supplementary file1 (DOCX 27 KB) [file 11356_2022_23625_MOESM1_ESM.docx]

**Supplementary Table 1. Association between metal levels in blood and TPOAb and TgAb among subgroups.**

| Reproductive-aged women  N=295 | Adjusted Model for TPOAb | | Adjusted Model for TgAb | |
| --- | --- | --- | --- | --- |
|  | OR (95%CI) | *P* | OR (95%CI) | *P* |
| Mg | 0.827 (0.746, 0.917) | <0.001 | 0.791 (0.696, 0.899) | <0.001 |
| Fe | 0.984 (0.977, 0.992) | <0.001 | 0.982 (0.973, 0.991) | <0.001 |

**Supplementary Table 1a**

**Supplementary Table 1b**

| Postmenopausal women  N=319 | Adjusted Model for TPOAb | | Adjusted Model for TgAb | |
| --- | --- | --- | --- | --- |
|  | OR (95%CI) | *P* | OR (95%CI) | *P* |
| Mg | 0.925 (0.842, 1.015) | 0.100 | 0.903 (0.822, 1.00) | 0.053 |
| Fe | 0.996 (0.987, 1.005) | 0.372 | 1.00 (0.991, 1.01) | 0.932 |

**Supplementary Table 1c**

| Men  N=468 | Adjusted Model for TPOAb | | Adjusted Model for TgAb | |
| --- | --- | --- | --- | --- |
|  | OR (95%CI) | *P* | OR (95%CI) | *P* |
| Mg | 1.06 (0.951, 1.19) | 0.279 | 0.951 (0.840, 1.08) | 0.433 |
| Fe | 0.997 (0.988, 1.01) | 0.476 | 1.01 (0.995, 1.02) | 0.284 |

Adjusted Model: This model is adjusted for age, BMI, HbA1c, TG, TC, alcohol consumption, current smoking and iodized salt intake. The menstrual history of 22 female was absent or not applicable.

OR, odd ratio; CI, confidence interval. BMI, body mass index; TPOAb, thyroid peroxidase antibodies; TgAb, thyroglobulin antibodies; TG, triglyceride; TC, total cholesterol.

**Supplementary Table 2. General Characteristics by Mg level quartiles.**

| Features | Mg level, mg/L | | | | *P* for trend |
| --- | --- | --- | --- | --- | --- |
|  | Quartile 1  <38.6 | Quartile 2  38.6-41.8 | Quartile 3  41.8-44.9 | Quartile 4  >44.9 |  |
| N | 278 | 276 | 275 | 275 |  |
| Age, year | 48.3 ± 14.9 | 50.6 ± 15.0 | 50.1 ± 14.0 | 50.3 ± 13.6 | 0.137 |
| Gender (male, %) | 24.8 | 36.6 | 46.5 | 61.8 | <0.001 |
| BMI, kg/m^2^ | 23.6 ± 3.83 | 23.7 ± 3.39 | 23.8 ± 3.37 | 24.8 ± 3.63 | <0.001 |
| HbA1c, % | 5.83 ± 1.08 | 5.90 ± 1.02 | 5.83 ± 0.783 | 5.85 ± 0.720 | 0.974 |
| TG, mmol/L | 1.08  (0.790, 1.55) | 1.19  (0.850, 1.72) | 1.24  (0.900, 1.97) | 1.39  (1.05, 2.08) | <0.001 |
| TC, mmol/L | 5.08 ± 0.938 | 5.45 ± 1.13 | 5.57 ± 1.26 | 5.71 ± 1.15 | <0.001 |
| HDL, mmol/L | 1.42 ± 0.330 | 1.42 ± 0.308 | 1.43 ± 0.342 | 1.39 ± 0.305 | 0.431 |
| LDL, mmol/L | 2.89 ± 0.791 | 3.22 ± 0.888 | 3.33 ± 1.01 | 3.51 ± 0.949 | <0.001 |
| Smoking, % | 12.2 | 19.6 | 23.7 | 26.5 | <0.001 |
| Alcohol, % | 5.00 | 5.10 | 10.1 | 8.60 | 0.034 |
| TSH, uIU/mL | 1.79  (1.24, 2.59) | 1.55  (1.07, 2.31) | 1.53  (1.04, 2.20) | 1.54  (1.07, 2.32) | 0.321 |
| FT4, pmol/L | 11.3  (10.3, 12.2) | 11.3  (10.3, 12.2) | 11.2  (10.3, 12.4) | 11.3  (10.1, 12.3) | 0.514 |
| FT3, pmol/L | 5.36  (4.85, 5.84) | 5.40  (4.93, 5.95) | 5.54  (5.12, 6.06) | 5.57  (5.09, 5.95) | 0.009 |
| TPOAb positivity, % | 24.8 | 14.9 | 14.9 | 12.4 | <0.001 |
| TgAb positivity, % | 19.4 | 13.8 | 11.6 | 9.8 | 0.001 |
| Iodized salt, % | 90.6 | 88.0 | 87.9 | 89.9 | 0.976 |

Continuous variables with normal distribution were presented as mean±SD, and with skewed distribution presented as median (interquartile ranges), respectively. Categorical variables were summarized as a numerical proportion. *P* values were calculated by Student’ s t test, Mann-Whitney U test and χ^2^ test. *P*<0.05 was considered as significant different.

BMI, body mass index; TPOAb, thyroid peroxidase antibodies; TgAb, thyroglobulin antibodies; TG, triglyceride; TC, total cholesterol; HDL, high-density lipoprotein; LDL, low-density lipoprotein; TSH, thyrotropin; FT3, free triiodothyronine; FT4, free thyroxine.

**Supplementary Table 3. General Characteristics by Fe level quartiles.**

| Features | Fe level, mg/L | | | | *P* for trend |
| --- | --- | --- | --- | --- | --- |
|  | Quartile 1  <468.4 | Quartile 2  468.4-506.34 | Quartile 3  506.35-543.15 | Quartile 4  >543.15 |  |
| N | 276 | 276 | 276 | 276 | - |
| Age, year | 51.2 ± 14.9 | 50.4 ± 15.2 | 50.4 ± 14.2 | 47.2 ± 13.1 | 0.002 |
| Gender (male, %) | 10.1 | 24.3 | 51.4 | 83.7 | <0.001 |
| BMI, kg/m^2^ | 23.4 ± 3.59 | 23.8 ± 3.62 | 24.1 ± 3.35 | 24.5 ± 3.71 | <0.001 |
| HbA1c, % | 5.89 ± 0.933 | 5.77 ± 0.684 | 5.82 ± 0.756 | 5.93 ± 1.19 | 0.462 |
| TG, mmol/L | 1.12  (0.820, 1.52) | 1.13  (0.800, 1.65) | 1.33  (0.935, 1.96) | 1.42  (0.963, 2.10) | <0.001 |
| TC, mmol/L | 5.24 ± 1.07 | 5.40 ± 1.21 | 5.59 ± 1.22 | 5.58 ± 1.01 | <0.001 |
| HDL, mmol/L | 1.44 ± 0.347 | 1.43 ± 0.297 | 1.41 ± 0.327 | 1.38 ± 0.310 | 0.010 |
| LDL, mmol/L | 2.98 ± 0.831 | 3.15 ± 0.972 | 3.38 ± 0.993 | 3.42 ± 0.883 | <0.001 |
| Smoking, % | 5.22 | 11.9 | 24.7 | 40.5 | <0.001 |
| Alcohol, % | 5.00 | 4.13 | 7.53 | 12.3 | <0.001 |
| TSH, uIU/mL | 1.71  (1.16, 2.53) | 1.56  (1.05, 2.48) | 1.62  (1.08, 2.52) | 1.47  (1.02, 2.10) | 0.004 |
| FT4, pmol/L | 11.2  (10.2, 12.2) | 11.5  (10.4, 12.3) | 11.4  (10.3, 12.5) | 11.2  (10.1, 12.2) | 0.985 |
| FT3, pmol/L | 5.28  (4.83, 5.75) | 5.43  (4.93, 5.93) | 5.46  (5.04, 5.89) | 5.70  (5.20, 6.23) | <0.001 |
| TPOAb positivity, % | 26.1 | 13.4 | 14.9 | 12.7 | <0.001 |
| TgAb positivity, % | 19.2 | 10.1 | 11.6 | 13.8 | 0.109 |
| Iodized salt, % | 89.7 | 87.7 | 91.6 | 87.2 | 0.792 |

Continuous variables with normal distribution were presented as mean±SD, and with skewed distribution presented as median (interquartile ranges), respectively. Categorical variables were summarized as a numerical proportion. *P* values were calculated by Student’ s t test, Mann-Whitney U test and χ^2^ test. *P*<0.05 was considered as significant different.

BMI, body mass index; TPOAb, thyroid peroxidase antibodies; TgAb, thyroglobulin antibodies; TG, triglyceride; TC, total cholesterol; HDL, high-density lipoprotein; LDL, low-density lipoprotein; TSH, thyrotropin; FT3, free triiodothyronine; FT4, free thyroxine.
